# Preliminary study to evaluate the phytochemicals and physiochemical properties in red and black date's vinegar

**DOI:** 10.1002/fsn3.1009

**Published:** 2019-04-29

**Authors:** Zeshan Ali, Haile Ma, Muhammad Tayyab Rashid, Asif Wali, Shoaib Younas

**Affiliations:** ^1^ School of Food and Nutrition Minhaj University Lahore Pakistan; ^2^ School of Food and Biological Engineering Jiangsu University Zhenjiang China; ^3^ Department of Agriculture and Food Technology Karakoram International University Gilgit Pakistan; ^4^ Department of Food Science and Nutrition University of Sargodha Sargodha Pakistan

**Keywords:** antioxidant activity, dates' vinegar, extraction solvent, physiochemical properties, total phenolic index

## Abstract

Antioxidant activity, total phenolic content (TPC), total flavonoids, carotenoids, pH, and total titratable acidity of red and black date's vinegar were analyzed. The extraction method was designed and optimized for this purpose with respect to the variety and solvent concentrations along with the time of ultrasonication. The results showed that red dates' vinegar has significantly (*p* < 0.05) higher total phenols (3.38 ± 0.13 mg GAE/ml) and antioxidant activity as compared to black dates' vinegar, which had a higher amount of carotenoids (3.43 ± 0.11 mg/100 ml). Similarly, red dates' vinegar has more flavonoids as compared to commercially available Zhenjiang vinegar. In terms of physiochemical properties, both red and black date's vinegar were not significantly different (*p* > 0.05). Use of 50% and 80% methanol with 25 min of ultrasonication for extraction seemed more effective. The total phenols, flavonoids, antioxidant activity, carotenoids, and physiochemical analysis of the red and black date's vinegar indicated that vinegar from dates (red or black dates) is a competitive product in the marketplace.

## 1 INTRODUCTION

Presently, consumer concern in healthy food is on the rise due to the advancement in the field of functional foods formulation. Moreover, consumers are demanding value‐added foodstuff with new attributes and this has led to several studies with the aim of optimizing health benefits of food products (Ali, Ma, Rashid, Ayim, & Wali, 2018; Ubeda et al., 2011). Various researchers have reported the association among the ingestion of vegetables and fruits in reducing the risk of cancer, cardiovascular disease, inflammation, and metabolic syndrome‐related complications (Hung et al., 2004).

Vinegar is a famous food product produced by a two‐stage fermentation technique from different kinds of raw fruits. Various researches conducted until now have reported fruit vinegar manufacturing through fermentation of pomegranates, apples, strawberries, and dates, and also from other uncommon raw materials, including fruit tree of Chinese litchi or Korean black raspberry (Ali et al., 2017; Nazıroğlu et al., 2014).

Most researchers have reported fruit vinegar to exhibit substantial antioxidative effect which aids in protecting functional organ against adversative outcomes of pathogenic flora. Fruit vinegar has been proven to have antidiabetic potential and reduce lipid concentration and blood pressure from carbohydrate food sources. Vinegar could be a potential source in the manufacturing of therapeutic preparations which is lesser in body straining (Ali, Ma, Wali, et al., 2018; Chou, Liu, Yang, Wu, & Chen, 2015; Dou, Li, Wang, & Cao, 2012). The robust antioxidative nature of vinegar is as a result of their bioactive, phenolic compounds and phytosterol compounds which are mainly characterized by tannins, phenolic acid, flavonoids, and anthocyanins (Charoenkiatkul, Thiyajai, & Judprasong, 2016).

Date, from the fruit of date palm, is an alternative of fruit source for vinegar production as a result of a range of essential nutrients with numerous latent health benefits. Dates are consumed either as dried or in the fresh form tamar (fully ripe) and rutab (semiripe) stages with no or little processing. In the processed form, they are transformed into jellies, jams, and syrup, and are utilized in various confectionary or bakery products together with honey, chocolate, coconut, vinegar, and others (Slobodníková, Fialová, Rendeková, Kováč, & Mučaji, 2016).

The main components of dates are carbohydrates (fructose, sucrose, and glucose), which may be composed of 70%. Dates' sugars are simply digested and can instantly be absorbed into the bloodstream after ingestion and metabolized to discharge energy for cell activities. Dates contain fiber vital mineral (iron, selenium fluorine, and calcium) and vitamins (Vayalil, 2012). Results have shown that extracts from dates have exposed free radical scavenging activity, immunomodulatory, and antimutagenic activities, (Allaith, 2008; Khan, Sarwar, Wahab, & Haleem, 2008) and dates have cardioprotective, anti‐inflammatory, antiobesity, and antihypertensive effects (Ali, Ma, Ayim, & Wali, 2018; Ali, Ma, Rashid, et al., 2018). In the past, dates' vinegar did not receive the attention it deserves due to limited literature about the fruit.

In the assessment of phytochemicals in agricultural products, various techniques available have been introduced prominent among them are the colorimetric assays. For colorimetric analysis to be possible, it is obligatory to have an extract or sample free of solid particles. The established method for the extraction of antioxidant compounds varies in few parameters in terms of solvent utilization. However, the key purpose of the extraction is to retain or extract much of the bioactive elements as possible (Saafi, El Arem, Issaoui, Hammami, & Achour, 2009). Earlier, researchers have reported the influence of various parameters (solvent type, ultrasonication time, percentage, and temperature) in the extraction of antioxidant compounds and phenolic compounds (Alothman, Bhat, & Karim, 2009; Spigno, Tramelli, & De Faveri, 2007). The purpose of this study was to evaluate the phytochemical and physiochemical properties of red and black dates' vinegar produced by solid‐state fermentation method. Comparison and correlation analysis of phytochemicals and antioxidants properties were done between various types of dates' vinegar. For this purpose, an extraction technique was designed in which variables (ultrasonication time and type of solvent) were optimized: Lastly, the values acquired from tested vinegar were compared with commercially available vinegar. To the best of our knowledge, this paper is the first to study the various phytochemical and physiochemical properties of red and black date's vinegar.

## 2 MATERIALS AND METHODS

### 2.1 Chemicals

Folin–Ciocalteu's reagent, acetone, ethanol, sodium dihydrogen phosphate monohydrate, anhydrous dipotassium hydrogen phosphate, anhydrous sodium carbonate, sodium acetate, potassium chloride, butylated hydroxyl toluene (BHT), and methanol were purchased from Merck (Darmstadt, Germany). 2, 2‐diphenyl‐1‐picrylhydrazyl (DPPH) free radical and ferric reducing antioxidant power assay (FRAP) reagent were purchased from Sigma‐Aldrich (Steinheim, Germany). Gallic acid and fluorescein sodium were provided by Fluka (Madrid, Spain).

### 2.2 Samples

Red and black date's vinegar was provided by Liyang Bask Vinegar Co., Ltd, Hebei, China. Dates were grown and harvested in Xinjiang, Hetian (China). Traditional solid‐state fermentation method was used to produce vinegar. The aging time of vinegar was more than 1 year at the time of analysis, while the Zhenjiang vinegar and persimmon vinegar were brought from the local market having more than 1‐year aging.

### 2.3 Extraction method

**Figure 1:**
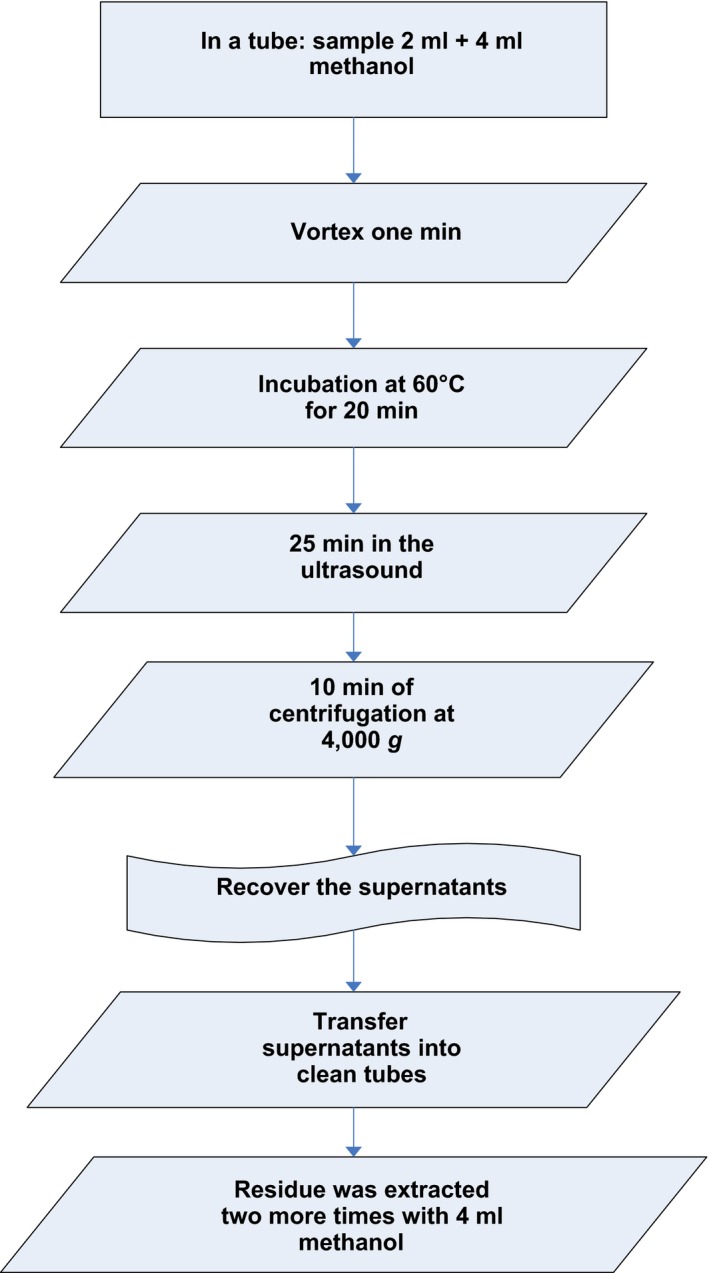
Extraction process

Because of dissimilar consistencies of the samples analyzed, it was essential to create an extraction method for the determination of antioxidant activity and total phenols. To establish the extraction system, little modification was made in the method of Chen, Fan, Yue, Wu, and Li (2008) and Gorinstein et al. (1999). Optimization of the utmost persuasive parameters in the extraction system was necessary; the parameters for optimization were a time of ultrasonication extraction (10, 15, and 25 min) and solvent percentage (50%, 80%, and 100%). The choice of superlative extraction parameters was made by taking into consideration the maximum values gained in every assessment plus economy of solvent used and time. The extraction method was displayed in Figure 1.

### 2.4 Determination of total phenolic content

Folin–Ciocalteu's assay (Singleton, Orthofer, & Lamuela‐Raventós, 1999) was used to determine the values of total phenolic content (TPC) through gallic acid as the standard. The mixture of sample (50 μl) solution, 3 ml distilled water, Folin–Ciocalteu's reagent solution (250 μl), and 750 μl of 7% Na_2_CO_3_ were mixed well and then incubated at room temperature for 8 min. 950 μl of distilled water was added. Afterward, the mixture was allowed to stand at room temperature for 2 hr and measured at 765 nm with distilled water as a blank. The TPC was measured as gallic acid equivalents (mg GAE/ml), and calibration curve linearity range was 50–1,000 μg/ml (*r* = 0.99).

### 2.5 Determination of radical DPPH scavenging activity

Chen and Ho (1995) method was used with slight modifications to determine DPPH free radical scavenging capacity of dates' vinegar. Concisely, 0.2 ml of sample was added to 3.8 ml ethanol solution of DPPH radical (0.1 mM). The mixture was left to stand for 30 min in dark after mixing well by vortex for 1 min. The absorbance of the sample (*A*_sample_) was determined against ethanol blank through the UV 160 spectrophotometer at 517 nm. Results were expressed and calculated as micromoles of Trolox equivalents (TE) per ml of dates' vinegar (mg TE/ml). 20–1,000 μM (*r* = 0.99) was the linearity range of the calibration curve.

### 2.6 Determination of FRAP

Szollosi and Varga (2002) method was used to determine FRAP assay, a recently prepared FRAP reagent warm at 37°C containing 25 ml of acetate buffer (0.3 µM pH 3.6), 2.5 ml of 10 µM TPTZ in 40 mM HCl, and 2.5 ml of 20 µM FeCl_3_·6H_2_O. Then, 50 µl of extract volume was mixed with FRAP reagent of 950 µl and measured at an absorbance of 593 nm for 5 min. Using an ascorbic acid standard curve, the FRAP value was uttered as AEAC (micromole ascorbic acid equivalent antioxidant capacity per milliliter [µmol AEAC/ml]).

### 2.7 Determination of total flavonoid content

Heimler, Vignolini, Dini, and Romani (2005) method was used to calculate the total flavonoid content (TFC). Concisely, 0.25 ml of the date's vinegar was extracted with 1.25 ml of distilled water, persuaded by adding 75 μl of 5% NaNO_2_ solution. 150 μl of 10% AlCl_3_·6H_2_O solution was added after 6 min and allowed to stand for another 5 min before adding 0.5 ml of 1 M NaOH. Then, 2.5 ml with distilled water was added in the mixture and mixed well and the absorbance was measured at 510 nm against distilled water as blank. The results were expressed and calculated as milligrams of Rutin (mg Ru/ml) by the calibration curve of Rutin. 10–1,000 μg/ml (*r* = 0.99) was the linearity range of the calibration curve.

### 2.8 Determination of total carotenoid contents

Sanusi and Adebiyi (2009) method was used to determine total carotenoids with slight modifications. Concisely, 0.5 ml of date's vinegar was extracted with 5 ml of ethanolic butylated hydroxyl toluene (ethanol/BHT—100:1, *v*/*w*) in triplicate for separation and the discharge of carotenoids. After mixing well, it was kept in a water bath for 5 min at 85°C. Then, for saponification 80% KOH (0.5 ml) was added and vortexed completely before placing it back for 10 min at 85°C in a water bath. 3 ml of cold deionized water was added followed by cooling down the mixture in an ice water bath. 3 ml of n‐Hexane was added to mixture before 2,500 × *g* centrifugation for two layers' separation to 5 min. The yellow colored upper layer was moved and collected. Until the upper layer become colorless, this method was repeated for four times. Hence, in each centrifuge tube, 12 ml of hexane was added and the end volume of every tube was noted. The measurement was done against the hexane as the blank at the wavelengths of both 450 and 503 nm. The results were given as mg/100 ml. The total carotenoids were calculated by Equation (1).

(1) $$\text{Total}\;\text{carotene}=4.642\times A_{450}-3.091\times A_{503}$$

### 2.9 Statistical analysis

One‐way analysis of variance (ANOVA) was used to analyze data by using statistical software Minitab version 17 (Minitab Inc., PA, USA). *p* ≤ 0.05 was considered to indicate a statistically significant variance. Analyses were conducted in triplicate, and results were reported as mean ± standard deviations.

## 3 RESULTS AND DISCUSSION

### 3.1 Extraction process optimization and solvent used

The criteria picked for optimization of the extraction parameters (type and percentage of solvent and time of ultrasonication) were the highest values of total phenolics, antioxidant activity, and solvent, and time‐saving. Selection of solvent is still an intricate issue despite the fact that it is probably the most investigated parameter. Resulting activities of antioxidant and extract yields of the sample are sturdily reliant on the extracting solvent nature. This is because of the existence of various antioxidant compounds of different polarities and chemical characteristics that may or may not be soluble in a specific solvent (Sultana, Anwar, & Ashraf, 2009). In our case, we used various percentages of methanol as a solvent for the extraction process to evaluate the best percentage of methanol. Sultana et al. (2009) reported that use of methanol as a solvent showed the best outcomes in the TPC determination (12.2 ± 0.28 mg GAE/ml) and TFC assay (8.66 ± 0.21 mg Ru/ml) determination, and they also explained that these values were based on the composition of different medicinal plants, and these types of plants comprise various compounds which offer antioxidant activity having various response and solvent affinity of the particular assay. Our outcomes are also in agreement with the preceding study of Chatha, Anwar, and Manzoor (2006), who stated that maximal extract yield (g/100 g) was gained with aqueous methanol from rice bran.

### 3.2 Solvent percentage effect

Various studies have proposed that the phenol recovery depends on the type of fruit and the percentage and kind of solvent used (Alothman et al., 2009). Hence, methanol was the leading solvent, and we used various percentages of methanol (50%, 80%, and 100%) to access the aqueous solution. Sultana et al. (2009) also showed the same results who analyzed methanol and its mixtures with water (80%) for therapeutic plants as the extraction solvent. Aqueous solution showed the higher content of phenols and enhanced antioxidant activities. In another study, a researcher showed that alcohol and water mixtures exhibited better recoveries of phenolic contents as compared to a solvent system based on monocomponent (Pinelo, Del Fabbro, Manzocco, Nuñez, & Nicoli, 2005). Similarly, Yilmaz and Toledo (2006) reported that in the grape seed powder ethanol extraction, an enhancement in the extracted phenolic content appeared when they increased the volume of water in the mixture (0%–30%) and phenolic content decreased at high percentages of water. The solvent‐to‐water ratio of 80:20 proved to be best for obtaining maximum values for most of the parameters analyzed.

### 3.3 Effect of ultrasonication time

**Figure 2:**
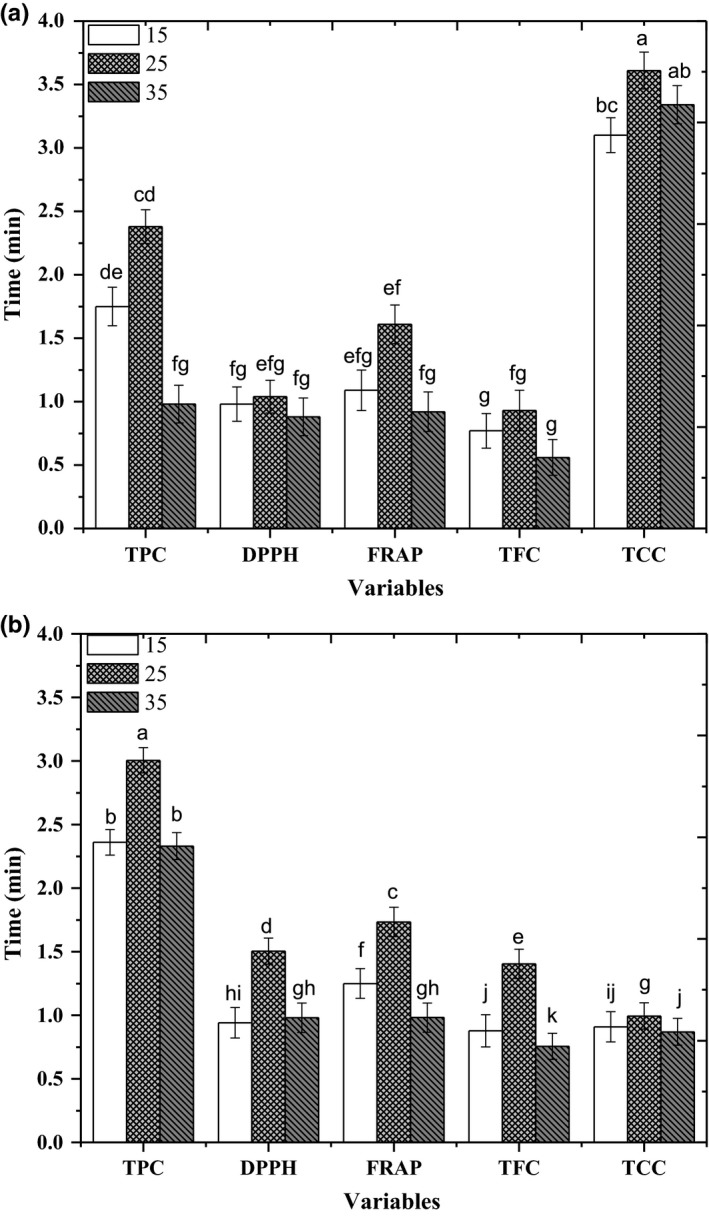
(a) TPC, DPPH, FRAP, TFC, and TCC values of red dates' vinegar for different ultrasound time tested. Values are presented as mean ± *SD*. (b) TPC, DPPH, FRAP, TFC, and TCC values of black dates' vinegar for different ultrasound time tested. Values are presented as mean ± *SD*. DPPH: 2, 2‐diphenyl‐1‐picrylhydrazyl; FRAP: ferric reducing antioxidant power assay; TCC: total carotenoid contents; TFC: total flavonoid content; TPC: total phenolic content

The acoustic cavitations and mechanical effects created in the solvent by the passage of an ultrasound wave permit for best diffusion of the solvent into the sample matrix (Lu & Luthria, 2016; Rostagno, Palma, & Barroso, 2003; Wang, Sun, Cao, Tian, & Li, 2008). Therefore, ultrasonication duration is a vital parameter for optimization. The best outcomes with reverence to antioxidant activity were gained using 25 min of ultrasonication, producing 1.34 ± 0.54 (µmol AEAC/ml) of FRAP and 1.04 ± 0.53 (mg TE/ml) of DPPH, respectively. Similarly, TPI values were also best extracted after 25 min of ultrasonication treatment. The TPC was 3.53 ± 0.31 (mg GAE/ml) at 25 min (Figure 2a,b). The outcomes agree with those preceding trials on the flavonoids extracted from plants. In one study, Zhang, Yang, Zhao, and Wang (2009) checked various extraction times (20, 25, 30, 35, and 40 min) and showed that flavonoid recovery reduced as a time of ultrasonication increased more than 25 min. Some researchers (Pinelo, Rubilar, Jerez, Sineiro, & Núñez, 2005; Spigno et al., 2007) have pointed out that incensement of temperature might denature few phenolic compounds; hence, this datum could elucidate the damage the activity of antioxidants too.

### 3.4 Total phenolic content

Phenolics apply their useful health outcomes majorly by their antioxidant activity (Fang, Yang, & Wu, 2002). These compounds are able of reducing the concentration of oxygen, precluding 1st‐chain initiation through scavenging initial radicals, crumbling initial products of oxidation to nonradical sorts, and breaking chains to avoid consistent hydrogen cogitation from substrates (Shahidi & Naczk, 2003). Phenolic compounds subsidize to the inclusive antioxidant activities of the foods originated from plants.

Total phenolic contents of the extracts from selected dates are shown in Table 1. Dates' vinegar from various extraction solvents varied significantly in their TPC (*p* < 0.05). The TPC of the red date's vinegar ranged from 1.89 to 3.05 mg GAE/ml, while the TPC of black date's vinegar ranged from 1.04 to 1.50 mg GAE/ml. The results proposed that 80% of methanol provide the maximum yields among the three different concentrations of solvent for extracting total phenols from red and black date's vinegar. This finding agreed well with the study of Ubeda et al. (2011), who found that maximum extraction of total phenols from persimmon vinegar was obtained by using 80% of ethanol. Similarly, one researcher showed the same results, who analyzed methanol and ethanol and their mixes with water (80%) as an extraction solvent for therapeutic plants (Sultana et al., 2009). Though there is not much research conducted on red and black date's vinegar, there are several types of research conducted on date fruits. From the outcomes of the total phenolic content of soft, semisoft, and dry dates, in comparison with semisoft and dry date vinegar, the soft date vinegar had the least total phenolic content (Hafzan, Saw, & Fadzilah, 2017). Mansouri, Embarek, Kokkalou, and Kefalas (2005) and Biglari, AlKarkhi, and Easa (2008) reported that TPC of Algerian and Iranian date palm fruit ranged, respectively, from 2.49 to 8.36 mg GAE/100 g of fresh and from 2.89 to 6.64 mg GAE/100 g of dry weight. These levels are almost similar to our results. In the other way, the study showed by Wu et al. (2004), on lipophilic and hydrophilic antioxidant capacities of common foods in the United States, found that Deglet Nour and Medjool varieties of dates offered a high level on total phenolic content (661 and 572 mg of GAE per 100 g FW, respectively) as compared to our study. Different factors such as dates' vinegar instead of dates' extract, growing condition, variety, season, maturity, fertilizers, geographic origin among two countries, amount of sunlight received, soil type, and experimental conditions (extraction, storage) among others might be responsible for observed variances.

**Table 1:** Effects of various percentages of methanol on phenols, antioxidants, and carotenoids

Sample (Methanol%)	TPC (mg GAE/ml)	DPPH (mg TE/ ml)	FRAP (µmol AEAC/ml)	TFC (mg Ru/ml)	TCC (mg/100 ml)
100% RDV	2.79 ± 0.11^a^	0.83 ± 0.04^c^	0.86 ± 0.03b^c^	0.67 ± 0.04^c^	0.88 ± 0.04^b^
80% RDV	3.38 ± 0.13^a^	0.98 ± 0.05^c^	1.10 ± 0.15^b^	0.95 ± 0.06b^c^	0.97 ± 0.04b^c^
50% RDV	2.57 ± 0.16^a^	1.16 ± 0.16^b^	1.36 ± 0.14^ab^	1.06 ± 0.12^b^	1.00 ± 0.08^b^
100% BDV	1.64 ± 0.15^b^	0.88 ± 0.03^c^	0.90 ± 0.06^c^	0.67 ± 0.03^d^	3.01 ± 0.13^a^
80% BDV	1.67 ± 0.13^b^	0.99 ± 0.02^d^	1.27 ± 0.15^c^	0.90 ± 0.05^e^	3.12 ± 0.15^a^
50% BDV	0.92 ± 0.14^d^	1.01 ± 0.14^c^	1.68 ± 0.15^b^	0.97 ± 0.04^cd^	3.43 ± 0.11^a^

Notes

BDV: black dates' vinegar; DPPH: 2, 2‐diphenyl‐1‐picrylhydrazyl; FRAP: ferric reducing antioxidant power assay; RDV: red dates' vinegar; TCC: total carotenoid contents; TFC: total flavonoid content; TPC: total phenolic content.

Data presented as mean ± *SD*. Values in the same row with different superscripted alphabet are significantly different at *p* < 0.05.

### 3.5 Radical DPPH scavenging activity

It is commonly accepted that free radicals formed in the body are partly correlated with the cancers etiology and other chronic ailments. Antioxidants from the diet, able of scavenging free radicals, are capable to decrease the disease risk. Hence, it is imperative to evaluate the radical scavenging effect of antioxidants in the date's vinegar. DPPH formed a violet solution in ethanol. Lessening of DPPH by antioxidants outcomes in deprivation of absorbance. Therefore, the marks of discoloration of the solution specify the scavenging efficiency of the added elements. The use of DPPH granted a rapid and easy way to determine antioxidant activity. The values of DPPH of red and black date's vinegar are given in Table 1. Dates' vinegar extracts from various percentages of extraction solvent varied considerably in their DPPH values (*p* < 0.05). The DPPH values of red dates' vinegar ranged from 0.77 to 1.18 mg TE/ml and black dates' vinegar from 0.64 to 1.01 mg TE/ml. The outcomes proposed that 50% and 80% of methanol and 25 min of ultrasonication were remained best for the evaluation of DPPH antioxidant assay from red and black date's vinegar. Similarly, results of one study showed that 25 min of ultrasonication along with 80% of ethanol was more effective for obtaining maximum values of DPPH scavenging activity from persimmon vinegar produced by various methods (Ubeda et al., 2011). Though there is not much research conducted on the antioxidant activity of red and black date's vinegar, there are several types of research conducted on date fruits. Saafi and his colleagues showed that Khouet Kenta dates showed the highest level of antiradical efficiency (1.96), while Allig showed the lowest level (0.72). The order of antioxidant activity of date palm fruit varieties based on DPPH method was as follows: Allig < Deglet Nour < Kentichi < Khouet Kenta. The outcomes showed that Algerian date palm fruit has a lower level of antioxidant as compared to Tunisia based on the same method. They stated that this variation is strongly related to the type of active compound and also to the polyphenolic content present in each variety (Saafi et al., 2009). These levels of DPPH values were almost similar to our results. The outcomes revealed that dates' vinegar either red or black is also free radical scavenger, mainly of peroxyl radicals, which are main diffusers of the oxidation chain of fat, thereby dismissing the chain reaction (Sakanaka & Ishihara, 2008).

### 3.6 Ferric reducing antioxidant power

Ferric reducing antioxidant power assay depends on the reduction of ferric tripyridyltriazine (Fe (III)‐TPTZ) complex to the ferrous tripyridyltriazine (Fe (II)‐TPTZ) via a reductant (different reducing agents or antioxidants) at less pH. Fe (II)‐TPTZ has a rigorous blue color. As compared to other tests of the total antioxidant assay, the FRAP assay is speedy, simple, highly reproducible, and inexpensive (Benzie & Strain, 1996). The FRAP values of antioxidant extracts from different percentages of extraction solvent varied considerably in their FRAP. The FRAP of red dates' vinegar ranged from 0.67 to 1.36 µmol AEAC/ml and black dates' vinegar values ranged from 0.72 to 1.31 µmol AEAC/ml. The outcomes proposed that 50% methanol was the best for determination of FRAP antioxidant assay from red and black dates' vinegar. Similarly, the results of different studies showed that the maximum antioxidant values (FRAP) were obtained by using methanol (Chatha et al., 2006; Sultana et al., 2009). Results revealed that red and black date's vinegar may serve as a good source of antioxidant. The capability of a methanolic extract of these dates to scavenge free radical (FRAP and DPPH) can be attributed to the existence of two major types of compounds. The first can be a wide range of phenolic compounds including caffeic, ferulic, gallic and sinapic acids, procyanidins, and flavonoids (Al‐Farsi, Alasalvar, Morris, Baron, & Shahidi, 2005; Hong, Tomas‐Barberan, Kader, & Mitchell, 2006; Regnault‐Roger, Hadidane, Biard, & Boukef, 1987) and the second the presence of other water‐soluble antioxidants such as oligo‐elements and vitamin C.

### 3.7 Total flavonoid content

Flavonoids are pervasive secondary metabolites of plants, containing flavonoids, flavones, and condensed tannins. Various researches proposed that the ingestion of foods rich in flavonoids defends against oxidative stress‐related human ailments. In vitro, flavonoids from different plant cradles have displayed free radical scavenging activity and defense against the oxidative strain. As components of fruits and vegetables, they are frequently encompassed in human food. Hence, there were no reports on quantification and identification of flavonoids in red and black dates' vinegar. In order to measure the latent role of flavonoids on antioxidant activity of the date's vinegar, total flavonoids of the extract were estimated and outcomes are presented in Table 1. Dates' extract from various extraction solvent percentages varied substantially in their TFC (*p* < 0.05). The TFC of red dates' vinegar ranged from 0.59 to 1.06 mg Ru/ml and black dates' vinegar values ranged from 0.48 to 0.97 mg Ru/ml. The outcomes proposed that 50% of methanol was the best between the three selected solvent percentages for extracting flavonoids from red and black date's vinegar. These findings agreed well with the study of Singh, Guizani, Essa, Hakkim, and Rahman (2012), who found that maximum extraction of total flavonoids from date's fruit was obtained by using methanol. He stated that total flavonoid content of date fruits varied noticeably from 19 to 66 mg in terms of catechin equivalents/ gm of DW of the sample. In general, higher flavonoid values were related to the rutab stage which specifies that the drying process may have a caustic effect on these compounds. Whereas positive significant correlations were noted between TFC and DPPH radical scavengers in the phytochemicals (Table 2).

**Table 2:** Pearson coefficient correlation of phytochemicals and antioxidants properties of methanol percentages

Variables	TPC	DPPH	FRAP	TFC	TCC
TPC	**1**				
DPPH	−0.0207	**1**			
FRAP	−0.5323	0.7049	**1**		
TFC	0.0200	**0.9343** *	0.7954	**1**	
TCC	**−0.9346** *	−0.0804	0.4138	−0.0733	**1**

Notes

DPPH: 2, 2‐diphenyl‐1‐picrylhydrazyl; FRAP: ferric reducing antioxidant power assay; TCC: total carotenoid contents; TFC: total flavonoid content; TPC: total phenolic content.

* Correlation is significant bold values at *p* < 0.05.

### 3.8 Total carotenoid content

Carotenoids are an enormous group of pigments that occur naturally in plants, different microorganisms, and algae. They are known to have a defensive effect on cardiovascular diseases, cancer, and chronic diseases. The chemical composition of carotenoids is generally based on a C40 tetraterpenoid structure with an extended conjugated, centrally located double‐bond system, which is linked to color presented, and acts as light‐absorbing chromophore (Meléndez‐Martínez, Britton, Vicario, & Heredia, 2007). Hence, there were no reports on quantification and identification of total carotenoid contents (TCC) in red and black date's vinegar. In order to measure the latent role of total carotenoids of the date's vinegar, total carotenoids of the extract were estimated and outcomes are shown in Table 1. Dates' extract from various extraction solvent percentages varied substantially in their TCC (*p* < 0.05). The TCC of red dates' vinegar ranged from 0.45 to 1.00 mg/100 ml and black date's vinegar values ranged from 1.48 to 3.47 mg/100 ml. The outcomes proposed that 80% of methanol was the best between the three selected solvent percentages for extracting carotenoids from red and black date's vinegar. Though there is not much research conducted in order to determine the total carotenoid content of red and black date's vinegar, there are several types of research conducted on date fruits. Results of one study showed that TCC in fresh dates Khasab, Fard, and Khalas were 1.31, 1.39, and 3.03 mg/100 g, respectively (Al‐Farsi et al., 2005). The highest total carotenoids in Khalas were predictable, as this variety has a yellow color, whereas the other two varieties are red. While in our study black dates' vinegar showed a higher level of carotenoids as compared to red, the outcomes of one study showed that the carotenoid content of freeze‐dried date was 0.22 mg/100 g of dry weight (∼0.18 mg/100 g of fresh weight) (Ben‐Amotz & Fishier, 1998). However, this is much lesser than those found in the present study, and this is probably due to the existing variance between the two samples in maturation, variety, analysis condition, and storage. In one study, Hart and Scott (1995) surveyed the carotenoid content of fruits and vegetables frequently consumed in the United Kingdom. In eight fruits, total carotenoid content ranged from 0.017 to 2.263 mg/100 g of fresh weight, being highest in mandarins and lowest in strawberries. Hence, dates' vinegar can be considered a good source of carotenoids compared to the above fruits. Further, negative correlations signify that TCC and TPC were not good radical scavengers between the phytochemicals (Table 2). The negative correlation between TCC and total phenolic content of date vinegar might be due to the existence of nonphenolic compounds in date vinegar that contribute to the total carotenoid property of date vinegar (Hafzan et al., 2017).

### 3.9 Physicochemical properties: pH, total sugar content, and titratable acidity

The total titratable acidity, pH value, and sugar content of date's vinegar are shown in Table 3. There was a slight variance in pH values of red and black date's vinegar, while there was a notable difference in pH values of dates' vinegar and commercially available Zhenjiang and persimmon vinegar. The pH of the date's vinegar varied from 2.73 to 2.69. The red and black date's vinegar have a higher content of total sugar as compared to Zhenjiang vinegar. While red dates' vinegar has more sugar than black date's vinegar. Soft cultivars were exposed to have a transformation of sucrose to fructose and glucose that instigated increase in total sugar of the fruit (Hafzan et al., 2017). Therefore, this could be clarified that red dates' vinegar formed from soft date cultivars had a higher content of sugar than black date's vinegar. The total titratable acidity of red (1.24%–1.86%) and black dates' (1.18%–1.81%) vinegar has a notable difference from commercially available Zhenjiang vinegar. This was because of extra acetic acid being added into commercial vinegar. In general, all of these physiochemical parameters are according to the results previously obtained by other authors in different kinds of vinegar (Hafzan et al., 2017; Sakanaka & Ishihara, 2008) and also according to international legislation.

**Table 3:** Physiochemical properties of red and black date's vinegar

Variable	Red dates' Vinegar	Black dates' Vinegar	Zhenjiang Vinegar
pH	2.71 ± 0.02^a^	2.70 ± 0.01^a^	2.69 ± 0.01^a^
Sugar content Brix *(%)*	13.37 ± *0.03^a^*	13.31 ± 0.05^a^	11.10 ± 0.00^b^
Total titratable acidity (%)	1.86 ± 0.03^b^	1.81 ± 0.04^c^	2.36 ± 0.04^a^

Note

Data presented as mean ± *SD*. Values in the same row with different superscripted alphabet are significantly different at *p* < 0.05.

### 3.10 Comparison with commercial vinegar

Zhenjiang and persimmon vinegar were picked from the local market to compare them with date's vinegar (Table 4). Results showed that the average antioxidant (DPPH) values of date's vinegar were lower than Zhenjiang (1.35 ± 0.14 mg TE/ml) and persimmon vinegar (1.12 ± 0.13 mg TE/ml), while the phenolic and carotenoids were higher. The phenolic and antioxidant activity values in dates' vinegar were related to those stated by preceding authors in various vinegar (Sakanaka & Ishihara, 2008). Similarly, Hafzan and his colleagues stated that pH values of commercial and homemade date vinegar varied significantly (*p* < 0.05). For homemade date vinegar, there was also a notable difference between soft and semisoft date vinegar. The pH of date vinegar varied from 2.77 to 2.77. These values were related to our outcomes. He also showed that semisoft date vinegar had a lower total sugar content as compared to commercial date vinegar (*p* < 0.05). (Hafzan et al., 2017). Further, no significant correlations were noted between the dates and commercially available vinegar (Table 5). More research is required to determine the reason why antioxidant values are comparatively lower than other vinegar. As compared to commercially available vinegar, both red and black date's vinegar are a competitive product.

**Table 4:** Comparison with commercially available vinegars

Sample	TPC (mg GAE/ml)	DPPH (mg TE/ml)	FRAP (µmol AEAC/ ml)	TFC (mg Ru/ml)	TCC (mg/100 ml)
Z R V	1.67 ± 0.13^a^	1.35 ± 0.14^b^	1.19 ± 0.15^c^	0.81 ± 0.03^d^	1.32 ± 0.14^b^
PV	1.32 ± 0.14^a^	1.12 ± 0.13^c^	1.14 ± 0.12b^c^	0.89 ± 0.05^d^	1.19 ± 0.14^b^
R D V	2.57 ± 0.16^a^	1.16 ± 0.16^c^	1.36 ± 0.14^b^	1.06 ± 0.12^d^	1.00 ± 0.06^e^
B D V	0.92 ± 0.04^d^	1.01 ± 0.14^c^	1.68 ± 0.15^b^	0.97 ± 0.04^cd^	3.43 ± 0.15^a^

Notes

BDV: black dates' vinegar; DPPH: 2, 2‐diphenyl‐1‐picrylhydrazyl; FRAP: ferric reducing antioxidant power assay; PV: persimmon vinegar; RDV: red dates' vinegar; TCC: total carotenoid contents; TFC: total flavonoid content; TPC: total phenolic content; ZRV: Zhenjiang rice vinegar.

Data presented as mean ± *SD*. Values in the same row with different superscripted alphabet are significantly different at *p* < 0.05.

**Table 5:** Pearson coefficient correlation of date's and commercially available vinegar

Variables	TPC	DPPH	FRAP	TFC	TCC
TPC	1				
DPPH	0.4231	1			
FRAP	−0.3234	−0.6898	1		
TFC	0.4486	−0.5971	0.5377	1	
TCC	−0.7255	−0.6439	0.8804	0.1199	1

Notes

DPPH: 2, 2‐diphenyl‐1‐picrylhydrazyl; FRAP: ferric reducing antioxidant power assay; TCC: total carotenoid contents; TFC: total flavonoid content; TPC: total phenolic content.

Correlation is significant at *p* < 0.05.

## 4 CONCLUSION

It is concluded that 80% of methanol for phenolic and 50% of methanol for antioxidant activity along with 25 min of ultrasonication were the best extraction conditions, between the variables examined to acquire the higher values of antioxidant activity and highest extraction of phenolic compounds. Comparing the two kinds of dates' vinegar, red dates' vinegar has higher values of antioxidant activity and total phenols, while the black dates' vinegar has higher total carotenoids values as compared to red dates' vinegar. Similarly, dates' vinegar has higher phenolic and carotenoids values as compared to some commercially available vinegar. Both red and black dates' vinegar were not significantly different in terms of physiochemical properties. These results proposed that dates' vinegar either red or black has beneficial health outcomes and might be a competitive product in the market.

## ETHICAL APPROVAL

This study does not involve any human or animal testing.

## CONFLICT OF INTEREST

The authors declare that they do not have any conflict of interest.
